# A systematic review of the perceptions of adolescents on graphic health warnings and plain packaging of cigarettes

**DOI:** 10.1186/s13643-018-0933-0

**Published:** 2019-01-17

**Authors:** Aaron Drovandi, Peta-Ann Teague, Beverley Glass, Bunmi Malau-Aduli

**Affiliations:** College of Medicine and Dentistry, James Cook University, Building 047, Pharmacy, 1 James Cook Drive, Townsville, QLD 4810 Australia

**Keywords:** Tobacco control, Public health, Youth, Health literacy

## Abstract

**Background:**

Graphic health warnings on tobacco packaging and the plain packaging of tobacco products are key tobacco control interventions. This systematic review investigates the perceptions of adolescents towards these packaging interventions.

**Methods:**

Published, original-research, English-language articles from 1 January 2000 to 1 September 2017 were identified through a systematic literature search of the PubMed, CINAHL, PsycINFO, Web of Science, and Scopus databases. Articles describing investigations into the perceptions of adolescents aged 11 to 19 years towards graphic health warnings and/or plain-packaged cigarettes were included in this review.

**Results:**

Nineteen articles, involving 15,935 adolescent participants, of which 72.85% were non-smokers or ex-smokers and 27.15% occasional or daily smokers, met the eligibility criteria. Graphic health warnings were perceived as more effective than text-only warnings, with warnings depicting lung cancer, and oral diseases being perceived as particularly effective. Health warnings increased viewer fear, anxiety, shock, and guilt and were considered effective in preventing non-smokers from experimenting with tobacco and prompting current smokers to quit. Plain packaging reduced the attractiveness and other positive attributes of cigarette packaging, with darker colours found to be the most effective. When used in combination, plain packaging increased the visibility of graphic health warnings, with participants also perceiving them as having an increased tar content and having more serious health risks, and increased thoughts of quitting amongst smokers.

**Conclusions:**

Graphic health warnings and plain packaging appear to increase adolescent awareness of the dangers of tobacco use. Further research into the most effective warnings to use in combination with plain packaging is needed to ensure the greatest reduction in tobacco use and prevent tobacco-attributable morbidity and mortality in this vulnerable population.

**Electronic supplementary material:**

The online version of this article (10.1186/s13643-018-0933-0) contains supplementary material, which is available to authorized users.

## Background

Tobacco use continues to be a major contributor to global morbidity and mortality, being responsible for an estimated 7 million deaths per year, and the attributable cause of death for over half of persistent tobacco users [1, 2]. Multiple forms of cancer and cardiovascular and respiratory diseases are the adverse outcomes of greatest concern, with their risk and severity being influenced by individual patient factors, alongside the cumulative exposure to carcinogenic constituents over the lifetime of a smoker [3, 4]. Therefore, initial tobacco experimentation and the development of nicotine addiction during the formative years when the brain is still maturing is linked not only to more significant risks to long-term health, productivity, and life expectancy, but also to a greater tendency to continue the addiction into adulthood [5, 6]. Physiological and sociological differences to adult populations increase the likelihood of addiction, where adolescents can experience significant peer pressure to experiment with drugs such as tobacco, which contributes to the majority of active adult smokers having started smoking during their teenage years [7, 8].

This issue is compounded by a long history of tobacco industry marketing tactics targeting adolescents and young adults in preference over older adults, as they are vital to the survival of the industry as the next ‘generation’ of smokers [9–12]. Whilst tobacco manufacturers have insisted that their packaging and other marketing techniques are meant only to retain brand loyalty amongst adult smokers, internal tobacco manufacturer documents show otherwise [9–12]. These targeted marketing strategies are the product of decades of research into attractive colours, shapes, logos, and descriptors meant to appeal to and attract adolescents and young adults, and create brand loyalty early in the life of a smoker [9–14]. The use of attractive packaging, filters, and variant descriptors such as ‘light’, ‘mild’, and ‘smooth’ have been shown to create misconceptions amongst both smokers and non-smokers on the relative safety of different cigarette brands and variants within brands [9–11].

In response to these marketing strategies, and to curb the use of tobacco amongst adolescents, there have been a range of interventions and programs implemented, including tax increases, banned point-of-sale advertising, mass media campaigns, and school- and parental-based educational programs. As part of the World Health Organization’s Framework Convention on Tobacco Control (FCTC), articles 11 and 13 relate to the packaging and labelling of tobacco products, and tobacco advertising, promotion, and sponsorship respectively [15]. These aim to guide FCTC signatories in removing misleading impressions created by tobacco marketing, advertising, and branding and to ensure the use of sufficiently sized text and pictorial health warnings, to inform and educate the public on the dangers of tobacco use [15].

Countries implementing these bans make tobacco packaging one of the last available methods for tobacco manufacturers to promote their products and differentiate them from competitor’s products [16, 17]. However, even this ‘last bastion’ for advertising is being increasingly controlled, through mandated pictorial and graphic health warnings, and the standardised (plain) packaging of tobacco products, first introduced in Australia in late 2012, and now present and planned for introduction in several other countries [18]. Reviews evaluating the effectiveness of these recent implementations of graphic health warnings (GHW) and plain packaging (PP) have been ongoing, with the growing body of international evidence supporting their use [18–22]. However, no review to date has focused on the effects of these interventions on adolescents. This systematic review therefore aims to assess the perceptions of adolescents towards graphic health warnings and plain packaging of cigarette packaging, which are aimed at reducing tobacco use amongst this vulnerable population. We had significant interest in identifying how younger persons perceive tobacco use as a measure of social standing, the potential for harm caused by tobacco use, and how these perceptions were influenced by the packaging of tobacco products. This review aimed to answer the question: *How does tobacco packaging and labelling influence adolescents’ perceptions of tobacco products?*

## Methods

This review was conducted as part of a larger research project, using a protocol that is not currently published. The PRISMA (Preferred Reporting Items for Systematic Reviews and Meta-Analyses) guidelines were used as a reporting guide for this systematic review [23] (see Additional file 1).

### Eligibility criteria

Eligible articles were those that gathered the self-reported perceptions of adolescents towards cigarette packaging which were either plain-packaged, displayed graphic health warnings, or both. These perceptions include any reported measure relating to perceived risks and attractiveness of packaging, as well as perceptions of the packs themselves, or smokers who use the packs. For this review, the relevant adolescent age was considered as being between the ages of 11 and 19 years old. This is the general age range of adolescents enrolled in middle school and high school, and where the use of tobacco generally becomes of concern within educational systems. Original-research articles published between 1 January 2000 and 1 September 2017, in the English language, were eligible for inclusion, whereas reviews, opinions, letters, and protocols were excluded. Articles which discussed the perceptions of young adults (18 to 35 years) or adults only were excluded, as well as those that did not differentiate data collected between different age groups if both adolescent and adult participants were enrolled. Other reasons for exclusion included the presentation and evaluation of text-only warnings on tobacco products, studies which did not gather self-reported adolescent participants’ perceptions (such as eye-tracking studies), studies that did not include GHW and PP perceptions as their primary outcome measure, and studies which asked participants to recall warnings they had seen in day-to-day life.

### Search strategy and study selection

Eligible articles were identified through a systematic literature search of the PubMed, CINAHL, PsycINFO, Web of Science, and Scopus databases. Searches utilised MeSH terms and combinations of the following words and their appropriate iterations: adolescent, perception, cigarette, plain packaging, graphic health warning, belief, behaviour, smoking, tobacco, warning, and young (see Additional file 2 for the detailed search strategy). Two authors (AD and BMA) were independently involved in article searching and screening and cross-checked each other’s final lists of eligible articles. Disagreements relating to article eligibility were resolved by consensus amongst all four authors. Titles were read to identify potentially relevant articles, and we initially included any article that appeared to present cigarette packaging to participants of any age or smoking status. Abstracts were reviewed, and articles which involved adolescent participants’ responses to cigarette packaging were retained, and those that matched the exclusion criteria were removed from the review. Eligible articles had their citations (using Google Scholar) and reference lists scanned to identify additional articles.

### Data extraction and quality appraisal

Data extraction was initially performed by a single author (AD), then independently cross-checked by a second author (BMA). Data extracted from eligible articles included author details, year published, country of participant origin, participant numbers and age range, gender distribution, smoking status, study design, interventions employed, and outcomes reported. The primary outcomes of interest for this review were the perceptions of adolescents towards cigarette packaging that displayed graphic health warnings, were plain packaged, or both. Responses gathered included ‘choice preferences’ and Likert-scale ratings of packaging attractiveness, perceived cigarette taste, perceived health risks, warning intensity, perceived smoker attributes, preferred pack selection, personal relevance of warnings, and perceived effectiveness in preventing smoking in non-smokers and prompting current smokers to quit. Study quality was assessed using validated checklists from the Joanna Briggs Institute (JBI). The JBI ‘Checklist for Analytical Cross Sectional Studies’ was used for 16 studies [24], and the JBI ‘Checklist for Randomized Controlled Trials’ was used for three studies [25]. These checklists assess for study clarity, appropriateness of methodological design, analysis, presentation of results, and alignment of results and discussion to research objectives.

### Data analysis

All outcome items were listed in a database, separated by type of intervention (GHW, PP, or both). Commonly described outcome items across the eligible articles (such as attractiveness of packaging for plain packaging studies, and perceived health risk across warnings for graphic health warning studies) were compared and reported relative to the intervention employed. Choice-based preferences and Likert-scale ratings which were identical or considered similar by authors (such as ‘appeal’ and ‘attractiveness’) were compared and pooled when describing the perceptions of adolescents to give clarity to the overall findings of each intervention type. Other findings relating to adolescent perceptions, such as the opinions of participants towards cigarette packaging warnings, were recorded separately and used to support the primary outcomes. The results of studies which did not receive a high quality score during the quality assessment were taken into consideration and are identified within the results.

## Results

### Study characteristics

**Fig. 1:**
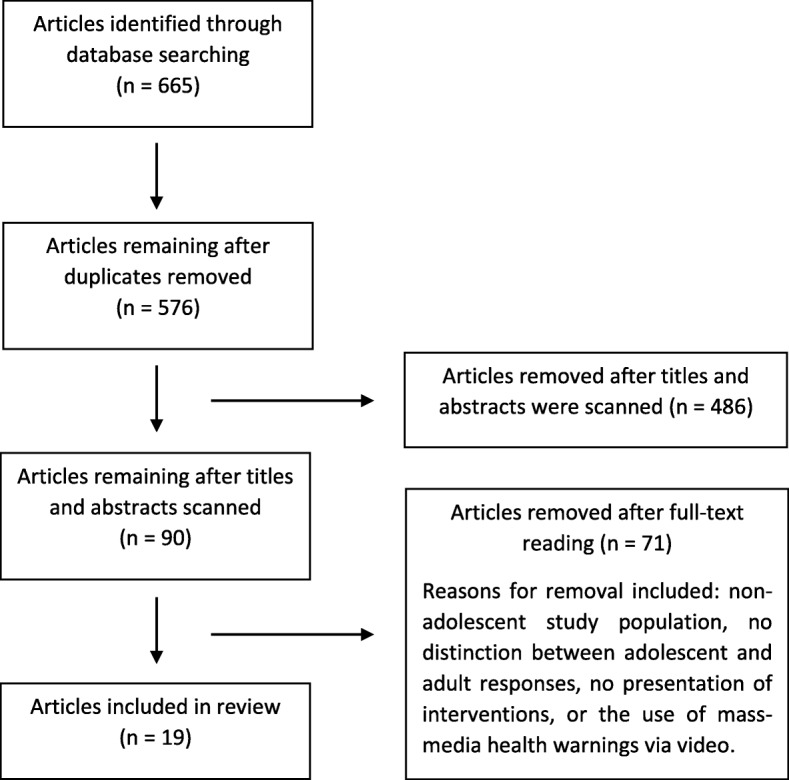
Flow chart of systematic literature search

Figure 1 illustrates the resulting number of eligible articles from the search strategy. The search strategy initially identified 576 potentially eligible articles (after duplicates were removed), which was reduced to 90 after abstract reading. Full texts were then read, resulting in a final number of 19 eligible articles. Common reasons for ineligibility were participant population being young adults, lack of distinguishing results between adolescents and older participants, queried participants on their perceptions without presenting interventional materials, displayed text-only warnings on cigarette packaging, or presented television/mass media warnings.

Table 1 details the study and participant characteristics of each article included in this review. A total of 15,935 participants were included in the 19 studies reviewed, 7267 (45.46%) of which were male, 8659 (54.58%) female, and 9 (0.06%) not-stated, all between the ages of 11 and 19 years. Nearly three quarters (72.85%) of participants were non-smokers or ex-smokers, and the remainder (27.15%) were occasional or daily smokers. Seven studies were conducted in Europe (*n* = 6150), one in Oceania (*n* = 1087), three in Asia (*n* = 4130), six in North America (*n* = 2958), one in Africa (*n* = 544), and one both in Europe and North America (*n* = 1066).

**Table 1:** Participant and methodological characteristics of articles eligible for inclusion in this systematic review (*n* = 19)

Year published and main author	Location, participant numbers, and age range	Gender distribution		Participant smoking status			Mode of study and interventions employed	Data collection and outcomes reported
		M%	F%	NS%	EX%	S%		
2009 Hammond [35]	UK *n* = 806 11–17 years	51.6	48.4	72.6	–	27.4	An online survey displaying six pairs of cigarette packs (using two brands), with branded, plain white, and plain brown packaging used, all displaying the same GHW.	Participants chose from each pair (or indicated ‘no difference’) which pack would have most tar delivery, smoothest taste, reduced health risks, highest attractiveness, and choice to smoke.
2009 Vardavas [26]	Greece *n* = 574 12–18 years	46.0	54.0	80.6	–	19.4	An in-school digital survey using computer-generated images, displaying pairs of seven existing text-only warnings with a comparative proposed GHWs on un-branded packaging.	Participants rated warnings using 5-point Likert scales on perceived effectiveness in preventing smoking, depicting the impact of smoking on health, and perceived warning strength.
2010* Fong [27]	China *n* = 396 13–17 years	50.8	49.2	87.9	8.1	4.0	Digitally constructed warnings were presented in person as photographs to adult and adolescent residents of four Chinese cities. Five pairs of cigarette packaging (four pairs with text-only versus GHW) were displayed.	Participants ranked and rated warnings using 5-point Likert scales on effectiveness in motivating smokers to quit, preventing youth smoking, informing the public on the harms of smoking, and showing government anti-tobacco initiative.
2010 Germain [42]	Australia *n* = 1087 14–17 years	49.4	50.6	60.4	21.9	39.7	An online survey, with each participant randomly viewing one of 15 packs, varying in brand presented (3 brands), degree of brand prominence, and size of GHW (3 × 5 design).	Participants rated on 5-point Likert scales; five perceived pack attributes, five perceived smoker attributes, and seven perceived cigarette attributes.
2011 Hammond [36]	USA *n* = 826 18–19 years	–	100	60.9	15.0	39.1	An online survey with participants viewing eight packages grouped into four categories: female-oriented brand with descriptors, female-oriented brand without descriptors, plain, and non-female-oriented brand.	Participants rated on 5-point Likert scales: brand appeal, brand taste, tar quantity, and health risks for each package. Participants also indicated on seven perceived attributes per pack (e.g. glamour, coolness, popularity) and their preferred pack.
2012a Hammond [28]	Mexico *n* = 528 16–18 years	50.0	50.0	51.1	–	48.9	Face to face survey with participants viewing warnings from 2 of 15 health-effect themes, each of which contained 1 text-only, and 4 to 6 pictorial warnings. Each theme included; graphic health warnings, lived experiences, symbolic representations, and testimonials.	Participants rated 11 measures on 10-point Likert scales, including perceived message: credibility, personal relevance, and affective responses. Four of these 11 items related to perceived effectiveness, including motivating smokers to quit and preventing non-smokers from smoking.
2012b Hammond [37]	UK *n* = 947 16–19 years	–	100	68.9	–	31.1	An online survey with participants assigned to one of four categories, each containing 10 cigarette packages: female-oriented brand with descriptors, female-oriented brand without descriptors, plain, and non-female-oriented brand.	Participants rated on 5-point Likert scales: brand appeal, brand taste, tar quantity, and health risks for each package. Participants also indicated on seven perceived attributes per pack (e.g. glamour, coolness, popularity) and their preferred pack.
2012 Moodie [38]	UK *n* = 658 10–17 years	47.3	52.7	90.9	–	9.1	An online survey with participants viewing several colours of plain cigarette packs with a text ‘Smoking Kills’ warning (white, red, green, light blue), and a brown plain pack of standard, sliding, and super-slim designs.	Participants rated the four coloured packs on 5-point Likert scales their perceived taste and harm. The standard brown plain pack was rated on eight perception items (four pack and four smoker items), and preference compared to other designs.
2013 Ford [39]	UK *n* = 1025 11–16 years	51.5	48.5	100	–	–	In-home surveys with participants viewing four branded packs (standard, slim, novel opening mechanism, and striking colour) and one plain pack with the same text warning.	Participants rated 11 items on 5-point semantic scales relating to package attractiveness, coolness, perceived harm, eye-catching, interest in smoking, and liking/disliking the pack.
2013a* Hammond [29]	USA *n* = 510 16–18 years	52.4	47.6	69.2	–	30.8	An online survey with participants randomly assigned to view two of nine sets of GHWs proposed by the FDA (6–7 warnings per set), with each GHW per set displaying the same text warning.	Participants rated several warning aspects on 10-point scales, including increase in concerns of health risks, efficacy motivating smokers to quit and preventing youth from smoking, and overall warning effectiveness.
2013b Hammond [43]	UK *n* = 762 11–17 years	54.9	45.1	93.8	1.0	4.9	An online survey with participants viewing six pairs of packs, comparing a regular pack to white or brown plain packs with moderate-sized text or graphic warnings (40%), or large-sized (80%) graphic warnings (2 × 3 model).	Participants selected from each pair (or indicated ‘no difference’) which pack would have most tar delivery, smoother taste, reduced health risks, highest attractiveness, would prompt to start smoking, and choice to smoke.
2013 Pepper [30]	USA *n* = 386 11–17 years	100	–	100	–	–	An online survey with participants randomly viewing one of four pack categories: addiction text-only warning, addiction text and image, lung cancer text-only warning, and lung cancer text and image (2 × 2 model).	Participants rated 5-point scales the perceived effectiveness of their warning in discouraging them from smoking, and the perceived likelihood and severity of suffering from the described condition (addiction or lung cancer).
2015* Alaouie [31]	Lebanon *n* = 1412 13–18 years	42.9	57.1	90.4% ex-smoker or non-smoker		9.6	Face-to-face interviews across 28 schools and universities, with students presented with two of five GHW on plain white packs compared to a locally available text-only warning.	Participants rated on 5-point Likert scales their perceived: message usefulness, noticeability, susceptibility, effectiveness, fear-arousal, self-efficacy in changing behaviour, intentions to not-smoke, and influencing family and close-contacts.
2015 Babineau [40]	Ireland *n* = 1378 16–17 years	55.7	43.7	78.6	4.2	17.2	In-school surveys for students across 27 schools. Pairs of packaging for three brands were presented. Packs were either branded or plain, with identical GHWs (lung damage).	Participants chose one pack (or indicated ‘no difference’) from each pair based on pack attractiveness, perceived health risks, perceptions of popular smoker attributes, and pack preference.
2016 Adebiyi [32]	Nigeria *n* = 544 13–17 years	44.7	55.3	98.3	–	1.7	In-school surveys in two schools in a single community, with participants viewing four GHWs: smoking harming children, and causing airway cancer, stroke, and impotence.	Participants indicated if each warning evoked: fear; shock, anxiety, or indifference. They also utilised a 3-point Likert scale on the effectiveness of each GHWs in preventing smoking initiation.
2016 Andrews [44]	USA, Spain, France *n* = 1066 13–18 years	50.0	50.0	–	–	100	An online survey with participants viewing one of eight packs (four plain and four branded) with varying levels of graphicness of GHWs, depicting the risks of smoking causing mouth cancer (2 × 4 model).	Participants rated using 6- and 7-point scales in response to the pack their: cigarette cravings, evoked fear (4 items), pack feelings (3 items e.g. embarrassed), and thoughts of quitting (4 items).
2016 Mutti [41]	Mexico *n* = 359 16–18 years	48.5	51.5	42.9	–	47.1	A face-to-face electronic survey with participants viewing a set of 12 gender-specific packs that were either fully branded or plain with brand name and descriptors.	Participants rated (yes/no/no difference) each pack on appeal, perceived taste, and perceived harm, with perceived smoker traits also rated (e.g. femininity, glamour, coolness, and popularity).
2016 Netemeyer [33]	USA *n* = 349 13–18 years	53.0	47.0	58.5	–	41.5	An online survey with participants randomly viewing one of nine cigarette packages containing a combined text and GHW.	Participants rated fear, guilt, and disgust evoked; perceived graphicness of the warning; and personal and perceived peer consideration of smoking after viewing.
2017 Reid [34]	India, Bangladesh, China, Korea *n* = 2322 16–18 years	50.2	49.8	77.3	–	22.7^^^	Online survey in Korea and China, and computer-assisted interviews in India and Bangladesh. Participants viewed 2 of 15 sets of cigarette packaging warning. Each set included 5–6 warnings on the same consequence of smoking, and included one text-only warning, GHW, lived experience, and testimonial.	Participants were assessed on their perceptions of the potential health effects of smoking for all 15 sets of warning after viewing their randomly assigned two sets. Participants either ‘agreed’, ‘disagreed’, or responded ‘do not know’ to each health consequence listed.

*GHW* Graphic health warning Alaouie et al. [31]: smoking prevalence higher in males (18.2% vs. 3.4%)—statistics do not include narghile smoking

*Adult smokers participated in this study, though their results have been omitted in this review

^There were significant differences in smoking status between different countries (see Table 2)

The 19 eligible studies used either face to face or electronic means to gather quantitative data from participants. This data included participant perceptions of a range of interventional materials involving cigarette packaging, including their perceptions of health risks and tar delivery, pack attractiveness, smoker attributes, pack attributes, personal relevance of warnings, and warning credibility. For the purposes of this review, pictorial and graphic health warnings, testimonials, and lived experiences will be grouped under and abbreviated as GHW, and plain packaging (including plain white and plain brown packs) will be abbreviated as PP. Nine studies evaluated perceptions towards different GHWs [26–34], seven evaluated perceptions towards branded versus PP cigarette packages [35–41], and three evaluated perceptions towards a combination of GHWs and PP [42–44].

### Quality appraisal

Sixteen studies were assessed by the JBI ‘Checklist for Analytical Cross Sectional Studies’ and scored out of eight, with four or below indicating low quality, five to six as moderate quality, and seven to eight as high quality [24]. Fourteen were found to be of high quality, and two of moderate quality [30, 32]. Three studies were assessed by the JBI ‘Checklist for Randomized Controlled Trials’ and scored out of 13, with seven or below indicating low quality, eight to ten as moderate quality, and ten and above as high quality [25]. All three RCTs scored were of high quality [42–44]. Table 2 details the quality appraisal outcomes of each study and the responses of participants to their respective interventional materials.

**Table 2:** Quality appraisal outcomes and study outcomes for each of the eligible studies (*n* = 19)

Year published and main author	Quality appraisal outcome	Intervention type* and analyses used	Key findings for adolescent perceptions of graphic health warnings and/or plain packaging^
2009 Hammond [35]	High (cross-sectional)	PP; chi-square, linear regression	• Both brands with plain white packs were perceived as less attractive, non-preferred, and having a lower tar content compared to the branded packs. • One pack brand was also considered as having a lower health risk, and one brand as having a less-smooth taste. • The plain brown packs were less attractive and less smooth for one brand, and less attractive, less smooth, higher risk, and non-preferred for the other brand compared to branded packs. All *p* values for these stated differences are < .001.
2009 Vardavas [26]	High (cross-sectional)	GHW vs. text warnings; chi-square, multivariate logistic regression	• GHWs were considered more effective than text-only warnings for 71.6 to 96.1% of participants, both in preventing non-smoking participants from smoking and in describing the effects of smoking on health. • Up to 84% of participants rated GHW as ‘effective’ or ‘very effective’ (4 or 5 out of 5) in preventing smoking initiation. • The GHW depicting lung cancer was rated as the most effective, followed by the GHW depicting foetal damage caused when smoking whilst pregnant. • Female participants had significantly higher effectiveness ratings of the GHWs depicting foetal damage, and protecting children from smoke (*p* < .05).
2010* Fong [27]	High (cross-sectional)	GHW vs. text warnings; chi-square, mixed-model ANOVA	• The four GHW packets were both rated and ranked as the most effective in motivating smokers to quit and preventing youth smoking, significantly higher than the six text warnings (*p* < .001), with the GHW depicting lung cancer rating the most effective, followed by the mouth disease, gangrene, and clogged arteries warnings (*p* < .05 between each warning). • The four GHW (with lung cancer as the highest rated) were also the most effective in informing the public on the dangers of smoking, with 81.5% of adolescents stating that packaging within China should contain more health information and 78.9% stating that packaging should include pictures instead of text-only warnings.
2010 Germain [42]	High (RCT)	GHW/PP; chi-square, ANOVA, principal component analysis	• Mean ratings of all positive pack, smoker, and cigarette attributes significantly reduced as branding and colour were progressively removed from packaging (*p* < .001), with ‘lower class’ perceptions concurrently becoming stronger (*p* = .043). • Smoking status was found to predict responses to pack ratings (*p* < .05), with established smokers having the most favourable perceptions of all packs. The addition of a larger GHW also had results dependent on smoker status, with experimenters and active smokers having the largest drop in perceptions of positive pack characteristics compared to susceptible and non-susceptible non-smokers (*p* < .01).
2011 Hammond [36]	High (cross-sectional)	PP; linear regression	• Compared to standard packs, of the eight brands used, plain packages were consistently the least appealing, were perceived as the worst tasting for six of the brands, had lower levels of tar for two of the brands, and were considered less harmful for two of the brands (all *p* < .05). • Plain packs also received significantly fewer positive ratings for every smoker trait (glamour, femininity, slimness, coolness, popularity, attractiveness, and sophistication) compared to standard packs (*p* < .001). • Significantly fewer participants preferred plain packs (*p* < .001).
2012a Hammond [28]	High (cross-sectional)	GHW; linear mixed effects models	• Text-only warnings were the lowest rated for all 15 health effects (*p* < .001), with the graphic warnings being rated as more effective than both the symbolic and lived experience warnings (*p* < .001), and those depicting external health effects perceived as more effective than those depicting internal health effects (*p* < .001). • Lived experience warnings that depicted effects on others were rated as more effective than those that depicted effects on oneself (*p* < .001), and susceptible non-smokers had significantly higher ratings than non-susceptible non-smokers (*p* = .02).
2012b Hammond [37]	High (cross-sectional)	PP; linear regression	• Plain packs received the lowest appeal (*p* = .013), and taste ratings (*p* = .027), were less likely selected as a preferred pack (*p* = .026), and were considered to have higher tar compared to the fully branded packs (*p* = .024). • Fully branded packs were also considered to have the lowest health risks compared to all other categories (*p* = .006). • For perceived smoker traits, plain packs received the lowest ratings for all seven attributes: femininity, slimness, glamorous, coolness, popularity, attractiveness, and sophistication (all *p* < .05).
2012 Moodie [38]	High (cross-sectional)	PP; chi-square	• Half of the participants associated colour and strength of taste, and colour and perceived harm, with the red pack considered the strongest tasting and most harmful and the light blue pack and white packs as weaker tasting and being the least harmful. • The brown plain pack was seen as largely unattractive, cheap, and uncool and used by boring, unfashionable, and older people. Smokers displayed less negativity towards the pack compared to non-smokers. • Smokers were more likely (*p* < .001) to prefer a pack, with the slide pack being the most popular of the brown plain packs.
2013 Ford [39]	High (cross-sectional)	PP; principal components analysis	• The mean ratings for all 11 items for all packs (e.g. attractiveness, coolness, harmfulness) were generally negative (none > 3 out of 5), with the plain pack being the most negatively rated, with mean scores ranging from 1.24 to 1.99 (*p* < .01). • The standard pack was also more negatively rated than the three novelty packs. • Unlike the branded packs, the plain pack showed no association between the 11 rated aspects, and smoking susceptibility.
2013a* Hammond [29]	High (cross-sectional)	GHW; linear mixed effects models	• Full-colour warnings were rated more effective than black and white warnings (*p* = .004), as were real people over comic book-style (*p* < .001), and those featuring quitline information (*p* < .001), particularly for current over non-smokers (*p* = .046). • Those with personal information were higher rated over those that did not (*p* < .004), as were those with graphic content compared to those that did not (*p* < .001), particularly for females over males. Mean scores were higher for ‘minority race respondents’ compared to ‘white respondents’ (*p* = .002).
2013b Hammond [43]	High (cross-sectional)	GHW/PP; chi-square, generalised estimating equation model	• Compared to branded packs, plain packs were considered less attractive, less likely to encourage smoking uptake, and had higher impact health warnings. Brown packs and those with graphic health warnings were also less likely perceived to have a smooth taste, present a lower health risk, or contain a lower amount of tar (all *p* < .001). • Larger GHWs were rated as the least attractive compared to moderate-size GHWs (*p* = .001) and text warnings (*p* < .001), were the least smooth tasting (*p* < .001 and *p* < .001 respectively), the least likely perceived to have a lower health risk (*p* < .001 compared to text warnings), the least likely perceived to have lower levels of tar (*p* < .001 and *p* < .001 respectively), and were perceived as having the highest impact on health (*p* < .001 and *p* < .001 respectively).
2013 Pepper [30]	Moderate (cross-sectional)	GHW; linear regression, ANOVA	• The lung cancer warnings (both text-only and text plus image) received higher ratings than the addiction warnings, with 60% of assigned participants rating them 5 out of 5 for discouraging smoking, compared to 34% for addiction warnings (*p* < .001). • There were no significant differences in deterring smoking or perceived risk for text vs. text plus image for either category. • Over half of assigned participants believed they would develop lung cancer if they smoked regularly, and over two thirds held this belief for developing nicotine addiction, with both categories also generally being considered as very severe.
2015* Alaouie [31]	High (cross-sectional)	GHW; McNemar test	• Participants perceived all GHWs as significantly more effective for all items compared to the text-only warning (*p* < .001). • Overall, compared to the text warnings, the lung cancer GHW received significantly higher effectiveness rating, followed by tooth decay, and death (all *p* < .01) except for female smokers due to low participant numbers. • All warnings were significantly more effective than text warnings (all *p* < .001) in preventing non-smokers from smoking.
2015 Babineau [40]	High (cross-sectional)	PP; chi-square, generalised estimating equation	• Two of the branded packs were perceived to be more attractive and healthier and used by ‘popular’ individuals, and were chosen twice as frequently compared to plain packs (all *p* < .001). • One pack brand (with pink and purple colouring) had a lower margin for choice (*p* < .001) and did not experience differences in attractiveness (*p* = .08), between the two packs, though the branded pack was perceived as healthier (*p* < .001). • Female participants were significantly more likely than males to associate this brand with popularity (*p* = .03).
2016 Adebiyi [32]	Moderate (cross-sectional)	GHW; bivariate analysis	• Responses to the four GHWs included fear in 37.3–56.4%, shock in 23.3–37.3%, anxiety in 2.9–21.1%, and indifference in 3.3–20.0% of participants. The GHW suggesting that smoking causes impotence had the highest indifference rating. • The GHW depicting airway cancer had the highest fear and shock ratings, and the lowest ratings for anxiety and indifference, and perceived as the most effective in preventing adolescents from smoking, especially those < 15 years (*p* < .05). • The GHW stating cigarette smoke harming children received the highest frequency of anxiety.
2016 Andrews [44]	High (RCT)	GHW/PP; multivariate analysis	• The two most graphic health warnings significantly increased thoughts of quitting, evoked fear, and reduced feelings towards the pack and cigarette cravings compared to the control and low-graphic health warning (all *p* < .05). • Plain packaging led to significant reductions in cigarette craving and feelings towards the pack (*p* < .05) and increased evoked fear (*p* < .05), but had no effect in increasing thoughts of quitting. • There were no combined effects overall for PP and GHWs, though there were some combined effects in France and Spain in reducing cravings and pack feelings respectively, though there were smaller cell sizes and reduced statistical power.
2016 Mutti [41]	High (RCT)	PP; chi-square, linear regression models	• Plain (with descriptor) packages received significantly lower ratings for appeal and taste (both *p* < .001) compared to branded packs, though there was no significant difference in perceptions of harm. • Female participants were more likely to give higher appeal and taste scores and rate packs as less harmful compared to males (*p* < .001, < .001, = .02 respectively). • Smokers were more likely to give higher taste ratings and consider packs as less harmful compared to non-smokers (*p* < .05). • Non-smokers rated branded packs significantly higher for all positive smoker-image traits (all *p* < .05), whilst smokers only rated two traits higher from branded compared to plain packs (stylish and sophistication, both *p* < .05). • Older adolescent participants also rated positive smoker-image traits higher than younger participants.
2016 Netemeyer [33]	High (cross-sectional)	GHW; linear regression models	• Perceived graphicness was associated with an increase in evoked fear and guilt (*p* < .01) for smokers and non-smokers. • Smokers had lower levels of disgust with increased graphicness compared to non-smokers. • Increased graphicness also led to increased hesitance (reduced personal consideration) towards smoking. • Stronger emotions in response to higher levels of perceived graphicness were more significant in smokers compared to non-smokers.
2017 Reid [34]	High (cross-sectional)	GHW; chi-square, ANOVA, logistic regression	• Perceptions of the health effects of smoking significantly increased for those who viewed the mouth cancer, heart disease, emphysema, and stroke (China and Korea), throat cancer (Bangladesh and Korea), skin ageing (India), impotence (India, China, and Korea), and gangrene (Bangladesh, India, and Korea) warnings (all *p* < .05). • Three quarters of participants in China, Bangladesh, and Korea and half in India also believed that cigarette packages should include more health-related information than the current packaging warnings were displaying in their respective country.

*GHW Graphic health warning (includes any form of pictorial warning, lived experience, and testimonials), *PP* plain packaging

^Results in these studies discussing adult participants, or adolescent perceptions of text-only warnings were excluded from this table

### Graphic health warnings

#### Graphic image versus text warnings

Nine studies in this review reported on adolescent perceptions on the effectiveness of text warnings compared to GHWs [26–28, 30, 31, 34] and/or between different GHWs on cigarette packaging [28–34]. GHWs were perceived as more effective than text warnings across most outcome measures in these studies. This included their ability to communicate the negative health effects of smoking [26–28, 30, 34], prevent non-smokers from smoking [26–28, 31], and motivate current smokers to quit [27, 28]. Two studies gathered specific reactions towards warning type, with graphic warnings considered more useful, credible, personable, and noticeable compared to text warnings and more capable in arousing fear and influencing a reader’s self-efficacy in changing their smoking behaviours and discussing smoking with others [28, 31]. One study found no difference in participants’ perceptions of text warnings vs. text plus pictorial warnings, though we considered the pictures used in the study as not being as graphic in comparison to warnings utilised in other studies [30]. This study also found that nearly half of participants did not believe that they would develop lung cancer if they became regular smokers, and nearly one third holding this belief relating to smoking and addiction. However, this study received a ‘moderate’ quality score during quality assessment, with issues such as ambiguity in the questions asked to participants potentially affecting the accuracy of these findings [30].

#### Standout and poorly rated graphic images

When comparing multiple GHWs, most studies identified that GHWs depicting respiratory or lung cancer were perceived as the most effective compared to other GHWs [26, 27, 30–32]. Studies that aimed to gauge specific reactions towards diseases portrayed in GHWs found that lung cancer and an increased perceived graphicness of warnings resulted in higher ratings for inciting fear, guilt, and shock [32, 33]. Other GHWs of note included those that were increasingly graphic, those depicting foetal damage caused when smoking whilst pregnant [26], and those depicting oral diseases [27, 31, 33]. Impotence was the least effective of four warnings in one study, receiving the highest ‘indifference’ ratings by participants [32]. Skin ageing was also poorly rated in one study, with participants in only one of four countries having an increased awareness of this consequence of smoking [34]. Studies comparing methods for delivering GHWs also found that colour warnings were perceived as more effective than black and white warnings, those depicting real people as having a greater impact than those that were symbolic or cartoon-styled, and those that included quitline information over those that did not [28, 29]. Graphic images were perceived as more effective than symbolic or shared lived experiences, and those that depicted external rather than internal health effects [44].

#### Influencing participant characteristics

Some studies found significant differences in participant perceptions related to demographic characteristics, namely age, gender, and smoking status. One study found that female participants had significantly higher ratings for the warnings depicting foetal damage when smoking, and protecting children from cigarette smoke [26]. One study found that younger participants (those under 15 years) experienced higher levels of fear and shock and would be less likely to smoke when shown a warning depicting airway cancer (though the results of this study should be interpreted with caution due to receiving a moderate quality score) [32]. Smokers in particular reported higher levels of guilt with increased graphicness compared to non-smokers, though had lower levels of disgust towards graphic warnings [33]. In the two studies that asked participants relating to their overall perceptions of health warnings on tobacco products, a majority (> 75%) in both studies indicated that cigarette packaging should include more health-related information, including the use of graphic images [27, 34].

### Plain packaging

#### Overall perceptions of plain packaging

Seven studies investigated adolescent participants’ perceptions of plain-packaged cigarettes, with most of the studies comparing white and/or brown plain-packaged cigarettes to fully branded, or partially branded packs (with or without accompanying health warnings) [35–41]. One study evaluated multiple colours of plain-packaged cigarettes [38], and one study evaluated plain-packaged cigarettes versus novelty branded cigarette packs [39]. In comparison to fully branded or partially branded packs, most of these studies identified that the brown-coloured, plain-packaged cigarettes were perceived by participants as having the lowest attractiveness/appeal, inferior taste, increased tar content, and an increased risk of causing ill-health [35–37, 39–41]. White packs were also perceived as less attractive and not preferred compared to branded packs in one study [35].

#### Impact of branding elements

Whilst some participants recognised that cigarette packaging does not influence health risk and tar delivery [35], a concerning theme which arose in some studies was the misperception that PP cigarettes had a lower tar content, reduced health risk, or were better tasting compared to branded cigarettes [35, 36, 38]. Colouration used when plain packaging cigarettes was found to be a critical aspect in one study, with half of participants associating the colour of the pack with cigarette harm and taste [38]. Whilst the brown plain pack was perceived as it was in other studies (unattractive, cheap, and uncool), the red pack was perceived as the strongest tasting and most harmful, whilst the white and light blue packs were perceived as being weaker tasting and the least harmful [35, 38]. However, one study found that for two of the brands presented, brown plain packs were perceived as having a reduced tar content and would cause less harm [35]. Smokers in one study also showed less negativity towards a brown plain pack compared to non-smokers [38]. Text descriptors on packaging (such as ‘smooth’ and ‘gold’) were also found to sometimes significantly influence participant perceptions when used on plain packs, perceiving them as containing less tar, having a lower health risk, and being more attractive [35].

#### Perceived pack and smoker attributes

Apart from comparisons of adolescent perceptions of cigarette quality and safety, several studies investigated perceptions of positive pack attributes, such as coolness, glamour, popularity, and femininity (for female participants). Akin to the perceptions of quality and safety, plain-packaged cigarettes were similarly the lowest rated for these measures compared to partially or fully branded packs [36, 37]. Perceived smoker attributes were also assessed in several of these studies, where participants rated their perceptions of a smoker of branded compared to plain-packaged cigarettes, with characteristics such as being cool, popular, attractive, and sophisticated being significantly lower than branded packaging [36–38, 40, 41]. Five studies also explored participants’ views on their preferred pack, and plain packs were consistently the least likely to be chosen compared to both standard and novelty branded packs [35–37, 39, 40].

#### Influencing participant characteristics

Female participants were more likely to associate a pink and purple branded pack with a positive smoker attribute (popularity) in one study [40] and gave higher appeal and taste scores and lower harm scores compared to males in another study [41]. This study also found that smokers gave higher taste ratings and considered smoking to be less harmful, whilst non-smokers gave significantly higher positive ratings for all smoker-image traits [41]. Older adolescents in this study also rated positive smoker-image traits [41].

### Combination of graphic health warnings and plain packaging

Three studies investigated adolescent perceptions of packaging with varied combinations of PP and GHW interventions [42–44]. Similar to the studies above evaluating the perceptions of either intervention used alone, GHWs increased perceptions of ill-health and thoughts of quitting, elicited fear, and reduced positive perceptions (such as attractiveness towards the pack), whilst PP also reduced packaging attractiveness, reduced intent to take up smoking, and affected perceptions of taste and tar content [42–44]. They also found that combining both types of intervention (the gradual removal of branding elements, and increased size or graphicness of GHW) led to further reduced positive pack perceptions [42, 43], and reduced cigarette cravings and pack attractiveness [44].

#### Influencing participant characteristics

Several perceptions were influenced by smoking status in two of the studies, whilst age and gender appeared to have no impact in any study. Smokers indicated higher positive perceptions towards all packs and a larger decrease in positive perceptions in response to large GHWs in one study [42], with another study’s smokers rating packs as more attractive and having a smoother taste than non-smokers [43]. One study reported that the American participants showed no significant differences in response to the combination of PP and GHW, whilst their French and Spanish counterparts indicated a reduction in cigarette cravings and pack attractiveness [44].

## Discussion

The objective of this systematic review was to identify and evaluate recent research investigating the perceptions of adolescents towards graphic health warnings and plain packaging of tobacco products. Participants in the 19 eligible articles generally perceived GHW as being effective in modifying their smoking behaviours and portraying the negative health effects of smoking compared to text warnings. PP was also perceived effective in contributing to an increased awareness of the health risks of smoking and reducing the attractiveness, popularity, and coolness of packaging and smoking. These findings support the position of the World Health Organization to ensure ‘consumers of tobacco products have a fundamental right to accurate information about the risks of smoking and other forms of tobacco use’ [45]. Adolescent risk perceptions differ from those of adults and may be more likely to engage in risky behaviours with the potential to have an adverse effect on personal health, stemming from a combination of targeted marketing and peer effects experienced during adolescence [7]. This emphasises the need for the development of tobacco packaging interventions to consider population differences, to ensure reductions in tobacco use amongst both adolescents and adults [7, 46].

The ‘Health Belief Model’ is a theoretical framework which predicts health-related behaviours (such as tobacco use) as being influenced by multiple internal and external factors, such as the perceived susceptibility and severity of tobacco-attributable diseases, benefits and barriers in modifying behaviours, and cues and self-efficacy in changing these behaviours [47]. Therefore, by minimising the attractive branding aspects of tobacco products, whilst simultaneously drawing attention to the health risks associated with tobacco use, GHWs and PP may act as prompts to quit amongst smokers, minimise the prevalence of experimental and daily tobacco use amongst adolescents, and the resulting continued use of tobacco into adulthood [7, 8].

In this review, pictorial health warnings were consistently perceived as more effective than text-only warnings in communicating the health risks associated with tobacco use and modifying non-smoker and smoker behaviours [26–28, 30, 31, 34]. This is supported by a recent meta-analysis that included both adults and adolescents, which reported that pictorial warnings attracted more attention, caused strong reactions, incited more negative attitudes towards packaging and smoking, and were more effective in reducing tobacco use [19]. The increased size and ‘graphicness’ (also referred to as strengthening) of health warnings has also been found to be an important aspect of individual warnings, resulting in improved knowledge of the risks of tobacco use and intentions to quit smoking [20]. In this review, GHWs depicting lung cancer were perceived by participants as being the most effective, followed by those depicting oral diseases [26, 27, 30, 32]. In comparison to text-only messages, GHWs which clearly depict negative (particularly external) health consequences of tobacco use have been theorised to have a greater public reach as they require minimal levels of health literacy for basic understanding. This is made more important by the trend of increased smoking prevalence amongst those with a lower level of education [48–50]. However, depicting short-term external health effects as opposed to longer-term chronic diseases may be more effective on adolescents, due to the ‘remoteness’ of conditions such as lung and mouth cancers [28, 51]. Further research is needed into the development of ‘ideal’ GHWs which can modify adolescent as well as adult perceptions and behaviours, especially considering some health effects in this review, such as skin ageing and impotence (believed to be very important to adolescents), were perceived as less effective than other GHWs [26, 32].

Similar to the findings in this review of the perceptions of adolescents towards plain packaging, a large systematic review (and a post-publication update) of both adolescents and adults identified significant reductions in packaging attractiveness as branding elements were removed [21, 22]. Perceptions of cigarette taste, safety, and quality and pack and smoker attributes were also consistent with the findings of this review [21, 22]. Though plain packaging was perceived as effective in influencing adolescent opinions of packaging and smoking when used alone, there were misperceptions identified amongst participants. Brightly coloured plain packaging can lead to perceptions of reduced tar content, reduced negative health consequences, and increased attractiveness of cigarette packaging [35, 38, 43]. Whilst the use of dark green/brown plain packaging initially implemented in Australia (and recently several other countries) may avoid this issue [18], some participants in one study perceived this colour as being less dangerous than branded packaging [36]. This emphasises the need for plain-packaged products to not only be dissuasively coloured, but also be accompanied by informative GHWs to ensure a reduction in pack attractiveness and increased perceived harm [36, 40]. The effects of PP regulations stem not only from its negation of attractive branding colours, but also via the removal of variant descriptors, meant to distinguish sub-types of cigarette products and attract and retain brand loyalty [9–11]. The banning of certain misleading descriptors such as ‘light’ and ‘mild’ has been an effective first step, though manufacturers have replaced these terms with others such as ‘smooth’ or ‘gold advance’, also capable of deceiving the public on the tar content, taste, and health risks of cigarettes [9–11].

Adolescent perceptions can be significantly influenced by demographic characteristics, such as smoking status, with several studies in this review reporting that current smokers (and to a smaller extent ex-smokers) were generally less affected by GHWs (and plain packs) compared to non-smokers [33, 38, 41, 42]. ‘Optimistic bias’ as described within these studies is a critical issue particularly amongst younger smokers, who believe themselves to be less vulnerable to the health consequences of smoking [33, 38, 41, 42]. As indicated earlier, future research should therefore focus on the development of targeted GHWs that can prompt cognitive reactions across a wide range of demographic profiles to facilitate the highest reduction in tobacco use. This was demonstrated in some of the included studies, such as female participants having higher perceived effectiveness ratings of foetal damage from smoking [26], and higher attractiveness ratings of ‘female-oriented’ packaging [40].

As adolescence is often a time for experimentation and risk-taking behaviours, during which there can be a quick loss of autonomy (with some researchers positing that this can occur after the first use of tobacco), reducing the attractiveness and glamour of tobacco packaging whilst highlighting the dangers is paramount [52–54]. With regard to message framing, loss-framed messages dominate mass media and packaging warnings, describing the negative consequences of smoking, whereas gain-framed messages describe the benefits of not smoking, or quitting. Whilst previous research has identified that graphic loss-framed warnings can have a higher rate of recall, some evidence suggests adult smokers experience greater reductions in tobacco use when shown gain-framed warnings [55, 56]. Research into adolescent reactions to loss- versus gain-framed messages would be ideal in ensuring the implementation of the most effective combination of GHWs and PP.

Apart from issues relating to misperceptions of warning irrelevance and optimistic bias amongst adolescents, a recent study investigating the 6-month, 2-year, and 5-year effects of GHWs found that though there was an increase in cognitive processing of warnings post-implementation, the 5-year survey found that there was a subsequent decrease back to pre-implementation levels [56]. This finding alongside similar findings in adult participants demonstrates that GHWs are most effective shortly after implementation but suffer from a loss of effectiveness over time, requiring a constant updating or rotation of warnings [56, 57]. It has also been suggested that PP would inhibit the loss of effectiveness of GHWs [57]. Two other studies have assessed the real-world impacts of PP alone on adolescents. One study found that only one fifth of adolescents had noticed PP nearly a year after implementation [58], whilst the other found that participants demonstrated an increase in support for PP, never-smokers reported they would be less likely to try smoking, and current smokers reported increased thoughts about quitting [59]. Whilst some results of these studies into the effects of GHWs and PP are promising, it is difficult to distinguish changes in responses pre- and post-implementation from concurrent trends in tobacco use and anti-tobacco interventions such as taxation policies and mass media campaigns.

Further research into the perceptions of adolescents in comparison to adults towards graphic health warnings and plain packaging is needed to identify the most effective combination of these interventions, especially when used alongside other interventions, such as mass media campaigns. School- and parental-based intervention programs, which focus on health risks associated with smoking displayed on cigarette packaging, may also be beneficial in reducing adolescent tobacco use [60].

### Strengths and limitations

The large number and geographical spread of participants included in this review allows for an increased generalisability of these findings across different populations and cultures and may be of relevance to many countries hoping to implement or update their anti-tobacco policies. This review also has several limitations, such as being unable to extrapolate the results to young adults, though similar in age, may undergo several perceptual changes secondary to their coming of legal age in purchasing tobacco. Their exposure to environments in which tobacco use is considered more socially appropriate compared to the school environment (e.g. workplaces, bars, and university) may also lead to altered perceptions. The use of electronic and internet surveys in many of the studies have their own limitations, such as preventing participants from viewing realistic 3D objects and facilitating tactile sensations, potentially not drawing a representative sample of the population, and having the perceptions given by adolescents potentially affected by nearby persons, such as their parents or teachers. A single exposure to the interventional materials in these studies is also a noteworthy limitation, as the responses given by participants may not be reflective of real-world conditions of multiple exposures after time and the potential for a stagnation of effects. Lastly, self-reporting bias was identified as a limitation in many of the included studies, where adolescents may report what they believe the researchers want to hear, rather than their true perceptions.

## Conclusion

Preventing tobacco use amongst adolescents and the resulting continued use into adulthood require the implementation of carefully designed and targeted anti-tobacco interventions. Dark-coloured packaging without branding elements and graphic health warnings depicting health consequences of smoking, such as lung cancer and oral diseases, appear to be perceived as more effective than bright-coloured packaging and those depicting other chronic tobacco-related issues respectively. As adolescents do not appear to perceive the threat of continued tobacco use in the same manner as adults, tailoring anti-tobacco interventions such as graphic health warnings and plain packaging towards this vulnerable population is essential in addressing adolescent tobacco use. Further research aimed at identifying the most concerning and emotion-responsive health conditions that could be depicted on packaging, in addition to plain packaging, would be a reasonable next step in anti-tobacco packaging interventions.

## Additional files

Additional file 1: PRIMSA checklist. (PDF 197 kb)

Additional file 2: Full search strategy. (DOCX 12 kb)

## Abbreviations

FCTC: Framework Convention on Tobacco Control
GHW: Graphic health warnings
JBI: Joanna Briggs Institute
PP: Plain packaging
PRISMA: Preferred Reporting Items for Systematic Reviews and Meta-Analyses
