# Supplementary material for: A systematic review of the perceptions of adolescents on graphic health warnings and plain packaging of cigarettes
Source: Syst Rev. 2019 Jan 17;8:25. doi: 10.1186/s13643-018-0933-0 (PMC6335796; doi:10.1186/s13643-018-0933-0)
Supplement: Supplementary file 2 — Full search strategy. (DOCX 12 kb) [file 13643_2018_933_MOESM2_ESM.docx]

General search terms utilised:

adolescent, perception, cigarette, plain packaging, graphic health warning, belief, behaviour, smoking, tobacco, warning, and young

**Pubmed search:** 1^st^ January 2000 to 1^st^ September 2017 date range

((adolescent[MeSH Terms]) AND percep*[Title/Abstract]) AND tobacco[Title/Abstract]

((adolescent[MeSH Terms]) AND percep*[Title/Abstract]) AND cigar*[Title/Abstract]

((adolescent[MeSH Terms]) AND percep*[Title/Abstract]) AND smok*[Title/Abstract]

((youth[MeSH Terms]) AND percep*[Title/Abstract]) AND tobacco[Title/Abstract]

((youth[MeSH Terms]) AND percep*[Title/Abstract]) AND smok*[Title/Abstract]

((youth[MeSH Terms]) AND percep*[Title/Abstract]) AND cigar*[Title/Abstract]

((adolescent behavior[MeSH Terms]) AND smok*[Title/Abstract]) AND warning*[Title/Abstract]

((adolescent behavior[MeSH Terms]) AND tob*[Title/Abstract]) AND warning*[Title/Abstract]

((adolescent behavior[MeSH Terms]) AND cigar*[Title/Abstract]) AND warning*[Title/Abstract]

((((adolescent behavior[MeSH Terms]) AND cigar*[Title/Abstract]) AND warning*[Title/Abstract] AND ( "2010/01/01"[PDat] : "2017/09/01"[PDat] ))) AND percep*[Title/Abstract]

(((adolescent behavior[MeSH Terms]) AND cigar*[Title/Abstract]) AND warning*[Title/Abstract] AND plain[Title/Abstract] ( "2010/01/01"[PDat] : "2017/09/01"[PDat] ))

((adolescent[MeSH Terms]) AND plain[Title/Abstract]) AND perception[Title/Abstract]

((adolescent[MeSH Terms]) AND plain packaging[Title/Abstract]) AND perception[Title/Abstract]

((adolescent[MeSH Terms]) AND graphic [Title/Abstract]) AND perception[Title/Abstract]

(((adolescent[MeSH Terms]) AND belief[Title/Abstract]) AND smok*) AND warning

((adolescent[MeSH Terms]) AND belief[Title/Abstract]) AND plain[Title/Abstract]
